# Circadian Clock Genes Regulate Temperature-Dependent Diapause Induction in Silkworm *Bombyx mori*

**DOI:** 10.3389/fphys.2022.863380

**Published:** 2022-04-27

**Authors:** Satoshi Homma, Akihisa Murata, Masato Ikegami, Masakazu Kobayashi, Maki Yamazaki, Kento Ikeda, Takaaki Daimon, Hideharu Numata, Akira Mizoguchi, Kunihiro Shiomi

**Affiliations:** ^1^ Faculty of Textile Science and Technology, Shinshu University, Ueda, Japan; ^2^ Graduate School of Science, Kyoto University, Kyoto, Japan; ^3^ Graduate School of Agriculture, Kyoto University, Kyoto, Japan; ^4^ Division of Liberal Arts and Sciences, Aichi Gakuin University, Nisshin, Japan

**Keywords:** clock gene, diapause, *Bombyx mori*, transcription activator-like effector nuclease (TALEN), circadian clock

## Abstract

The bivoltine strain of the domestic silkworm, *Bombyx mori*, exhibits a facultative diapause phenotype that is determined by maternal environmental conditions during embryonic and larval development. Although a recent study implicated a circadian clock gene *period* (*per*) in circadian rhythms and photoperiod-induced diapause, the roles of other core feedback loop genes, including *timeless* (*tim*), *Clock* (*Clk*), *cycle* (*cyc*), and *cryptochrome2* (*cry2*), have to be clarified yet. Therefore, the aim of this study was to elucidate the roles of circadian clock genes in temperature-dependent diapause induction. To achieve this, *per*, *tim*, *Clk*, *cyc*, and *cry2* knockout (KO) mutants were generated, and the percentages of diapause and non-diapause eggs were determined. The results show that *per*, *tim*, *Clk*, *cyc*, and *cry2* regulated temperature-induced diapause by acting upstream of cerebral γ-aminobutyric acid (GABA)ergic and diapause hormone signaling pathways. Moreover, the temporal expression of the clock genes in wild-type (*wt*) silkworms was significantly different from that of thermosensitive transient receptor potential ankyrin 1 (TRPA1) KO mutants during embryonic development. Overall, the findings of this study provide target genes for regulating temperature-dependent diapause induction in silkworms.

## Introduction

The circadian clock is involved not only in the daily regulation of several physiological, metabolic, and behavioral activities, including egg hatching and adult eclosion, but also in time-compensated Sun compass navigation, photoperiodism, and dormancy in insects (Patke et al., 2020; Saunders, 2020; Ahmad et al., 2021; Brady et al., 2021). Certain clock gene members drive the core functioning of the circadian clock, forming a transcriptional feedback loop module referred to as the “core feedback loop” (Hardin, 2011). An insect circadian clock model has been developed in *Drosophila melanogaster*; the first clock gene *period* (*per*) identified in *D. melanogaster* mutants reared in constant darkness (DD) allows free-running circadian rhythms (Konopka and Benzer, 1971). Previous studies have demonstrated that the core feedback loop is composed of products of *per*, *timeless* (*tim*), *Clock* (*Clk*), and *cycle* (*cyc*) in *D. melanogaster* (Hardin, 2011; Patke et al., 2020). CLK and CYC heterodimers drive *per* and *tim* transcription through E-box enhancer elements. The resultant PER and TIM proteins form heterodimers that translocate back into the nucleus to repress their own transcription *via* inhibitory effects on CLK and CYC, resulting in circadian oscillation in the expression of several clocks and related genes. Moreover, *Drosophila* cryptochrome (*Drosophila*-type CRY is designated as CRY1) is co-localized in clock cells with PER and TIM and functions as a blue-light photoreceptor involved in photic entrainment. CRY1 disrupts PER and TIM heterodimers by directly interacting with TIM in a light-dependent manner and also participates in its own light-dependent degradation (Hardin, 2011; Patke et al., 2020)^.^

Apart from CRY1, a vertebrate-like light-insensitive CRY designated as CRY2 has been identified in every non-drosophilid insect (Reppert, 2007). The monarch butterfly, *Danaus plexippus*, possesses two CRY proteins, one ortholog of the light-sensitive CRY1 and a second light-insensitive CRY designated as CRY2 (Yuan et al., 2007). CRY2, a clock factor that forms the core feedback loop, is the major transcriptional repressor of CLK/CYC-mediated transcription, whereas PER is associated with CRY2 stabilization rather than with active transcriptional repression (Reppert, 2007; Zhu et al., 2008; Zhan et al., 2011). These findings suggest that extensive functional diversity exists among species with regard to *cry* genes and that clock genes participating in the core feedback loop have been evolutionally selected in insects (Reppert, 2007; Tomioka and Matsumoto, 2015; Beer and Helfrich-Forster, 2020; Brady et al., 2021).

Over the years, clock genes, including *per*, *tim*, *Clk*, *cyc*, *cry1,* and *cry2*, have been identified in the domestic silkworm, *Bombyx mori* (Kawamoto et al., 2019). The expression of *per*, *tim*, *Clk, cry1*, and *cry2* exhibits temporal changes during embryonic development under both light/dark and DD conditions (Tao et al., 2017; Ikeda et al., 2019; Nartey et al., 2021). Additionally, core clock gene mutations disrupt not only the daily rhythm in both egg-hatching and adult eclosion rhythms in each *per* and *tim* knockout (KO; Ikeda et al., 2019; Nartey et al., 2021) but also the photoperiodic diapause, as a specific subtype of dormancy disrupted in *per* KO (Ikeda et al., 2021). Overall, research findings suggest that these clock genes operate as components of the core feedback loop module in the circadian clock of *B. mori*.

The bivoltine Kosetsu *B. mori* strain exhibits embryonic diapause, which is induced transgenerationally as a maternal effect (Yamashita and Hasegawa, 1985; Denlinger et al., 2012; Yokoyama et al., 2021). Maternal environmental conditions during embryonic and larval development influence progeny diapause. Generally, the temperature of the maternal environment is the predominant factor determining the occurrence of embryonic diapause, regardless of the photoperiod during embryonic and larval development (Shiomi et al., 2015; Tsuchiya et al., 2021). When eggs are incubated at 25°C under continuous darkness (25DD), the resultant female moths lay nearly 100% diapause eggs. In contrast, incubation of eggs at 15°C under dark conditions (15DD) resulted in moths that laid nearly 100% non-diapause eggs, although photoperiodic conditions during larval stages affected diapause determination when eggs were incubated at 20°C under continuous light (Ikeda et al., 2021). Thus, Kosetsu is considered a typical strain that is susceptible to temperature-dependent diapause induction.

The molecular mechanisms underlying the induction of embryonic diapause in *B. mori* have been extensively investigated (Denlinger et al., 2012; Tsuchiya et al., 2021). Our previous study revealed that embryonic diapause is induced by the diapause hormone (DH) signaling pathway, which consists of a highly sensitive and specific interaction between the DH and DH receptor (DHR) during pupal-adult development (Shiomi et al., 2015). DH is released from the corpus cardiacum (Sato et al., 1998) into the hemolymph and acts on DHR in the ovary (Homma et al., 2006). Moreover, cerebral γ-aminobutyric acid (GABA)ergic and corazonin pathways modulate DH release during temperature-dependent expression of the plasma membrane GABA transporter [GAT] (Tsuchiya et al., 2021). Recent studies revealed that the transient receptor potential ankyrin 1 (TRPA1; BmoTRPA1) acts as a thermosensitive channel activated at temperatures above 21°C and affects diapause induction through DH release in Kosetsu. Thus, BmoTRPA1 acts as a molecular switch for temperature-dependent diapause induction in Kosetsu (Sato et al., 2014; Yokoyama et al., 2021). However, it is unknown whether clock genes are involved in temperature-dependent diapause induction.

Therefore, the aim of this study was to elucidate the involvement of clock genes in temperature-dependent diapause induction. To achieve this, we generated KO mutants of the clock genes and examined their expression and effects on diapause induction.

## Materials and Methods

### Silkworms

The bivoltine Kosetsu *B. mori* strain was used in this study. Kosetsu exhibits a typical temperature-dependent diapause induction under rearing conditions (Tsuchiya et al., 2021; Yokoyama et al., 2021). The eggs were incubated under two different conditions: 1) at 25°C under continuous darkness (25DD) to obtain diapause eggs from wild-type (*wt*) silkworms and 2) at 15°C under continuous darkness (15DD) to obtain non-diapause eggs from *wt*. Larvae were then reared under a 13-h light/11-h dark cycle (13L11D) at 25°C as previously described (Shiomi et al., 2015). The pupae used in this experiment were collected within 1 h after pupation (referred to as day 0) to synchronize their subsequent development. The pupae were kept at 25°C under 12-h light/12-h dark (12L12D) conditions (Shiomi et al., 2015). The percentages of diapause eggs laid by the adults were estimated by counting the number of eggs in diapause after non-diapause eggs hatched. The results are expressed as the average percent diapause in each egg batch (Shiomi et al., 2015).

### KO Mutants

KO mutants of *per* (*per* ^*+13*^) and *BmoTRPA1* (*∆TRPA1_1,429*) were constructed following previously described procedures (Ikeda et al., 2021; Yokoyama et al., 2021). The transcription activator-like effector nuclease (TALEN)-based mutant lines of five genes (*tim*, *Clk*, *cyc*, *cry1*, and *cry2*) were constructed according to previous methods (Takasu et al., 2014; Yokoyama et al., 2021). DNA constructs containing TAL segments were prepared using a Golden Gate TALEN kit (Addgene, Cambridge, MA, United States). To screen for germline mutagenesis, the G_0_ adults were mated with *wt*. The oviposited G_1_ eggs were collected, and approximately 10 eggs from each brood were pooled for genomic DNA extraction using DNAzol reagent (Thermo Fisher Scientific, Waltham, MA, United States). DNA fragments containing the targeted region of interest were amplified by PCR using Takara Ex Taq (Takara, Tokyo, Japan) and specific primers (Supplementary Table S1). To test for mutagenesis, each PCR product of *tim*, *Clk*, *cyc*, *cry1*, and *cry2* was digested using the restriction enzymes *Sty*I, *Bsa*I, *Nco*I, *BsrB*I, and *Sal*I, respectively. Mutated PCR products were verified by sequencing. The broods containing mutated sequences were reared, and mutated G_1_ adults were crossed with siblings carrying the same mutation. Homozygous and hemizygous mutants were obtained after confirmation by sequencing the target region in the G_2_ or G_3_ egg genome.

### Picrotoxin and DH Injection

The plant alkaloid picrotoxin (PTX; 5 μg/μl; Sigma-Aldrich, St. Louis, United States), which is a widely used ionotropic GABA and glycine receptor antagonist (Masiulis et al., 2019), was dissolved in distilled water, and 10 µl was injected into pupae through the intersegmental membrane between the second and third abdominal segment at 1–6 h after pupation. DH of 95% purity (HPLC area percentage) was obtained from Operon Biotechnologies (Tokyo, Japan) and dissolved in peanut oil (Sigma-Aldrich); 10 µl solution at 10 pmol/μl was injected into the pupa, 3.5 days after pupation.

### DH Level in Hemolymph

The hemolymph was collected at ZT6 (ZT = zeitgeber time, ZT = 0 corresponds to lights on). The DH extract was prepared from the hemolymph as described previously (Shiomi et al., 2015). DH levels were measured using a time-resolved fluoroimmunoassay as described previously (Shiomi et al., 2015).

### Quantitative RT-PCR (qPCR) Analysis

The eggs were incubated at 25°C under 12L12D, entrained to 12L12D, and collected at 6-h intervals for a 1-day cycle (ZT0–24) and the following day in DD (circadian time; CT0∼24). The eggs were collected at the appropriate time point and stored at −80°C until RNA extraction. RNA extraction and first-strand DNA synthesis were performed using TRI Reagent^®^ (Molecular Research Center, Inc, Cincinnati, OH, United States) and ReverTra Ace^®^ qPCR RT Master Mix with gDNA Remover (TOYOBO, Osaka, Japan) containing the random primers and oligo dT primer, respectively. qPCR analysis was performed for the four sets of eggs using TB Green Premix DimerEraser using the Thermal Cycler Dice^®^ Real-Time System (TP800; Takara Bio, Tokyo, Japan) and specific primers shown in Supplementary Table S1. The qPCR conditions were as follows: 95°C for 30s and then 40 cycles of 95°C for 5 s, 55°C for 30 s, and 72°C for 30 s with 0.3 µM concentration of each primer. The efficiency of amplification and detection by all primer sets was validated by determining the slope of Ct versus dilution plot on serial dilution. Individual reactions were used to quantify each RNA level in a given cDNA sample, and the average Ct from four samples within the same run was used for quantification. For statistical analysis, the data for each gene were normalized to *rp49* as an external standard and normalized to the average of all time points within a set.

### Statistical Analysis

Statistical parameters, including definitions and values of *n*, are provided in the relevant figures or corresponding figure legends. Statistical analyses were performed in Excel 2011 (Microsoft) using software add-in Toukei-Kaiseki Ver. 3.0 (Esumi). *p*-values were determined using the Steel–Dwass test in diapause-egg-inducing activity and by one-way analysis of variance (ANOVA), Tukey–Kramer, and Student’s *t*-tests in temporal expression change in qPCR analysis and others. Data are expressed as mean ± standard deviation (SD). The mean values were considered to be significantly different at *p < 0.05, **p < 0.01, and ***p < 0.001; ns indicates no significant difference.

## Results

### Construction of Core Feedback Loop Clock Gene KO Mutants

**FIGURE 1:**
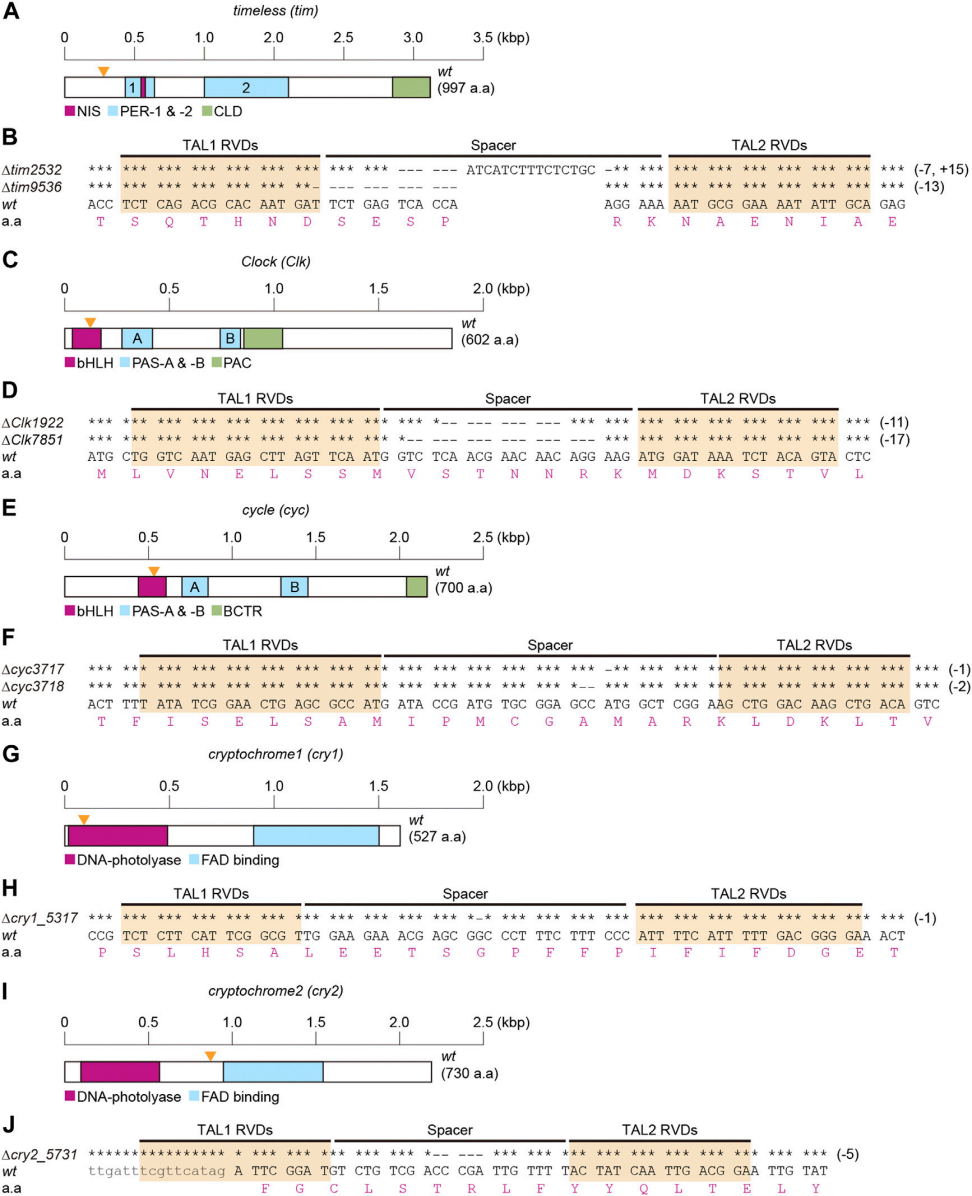
Construction of clock gene knockout mutants. Clock genes include *timeless* [*tim*] **(A,B)**, *Clock* [*Clk*] **(C,D)**, *cycle* [*cyc*] **(E,F)**, *cryptochrome1* [*cry1*] **(G,H)**, and *crtyptochrome2* [*cry2*] **(I,J)**. Schematic representations of the coding regions of each cDNA structure are shown in **A,C,E,G,I**. **(A)** Functional domains of TIM, NIS (nuclear localization signal), PER (PER dimerization domain)-1 and -2, and CLD (cytoplasmic localization domain) are represented as referred in Iwai et al. (2006), and Tomioka and Matsumoto (2015). **(C)** Functional domains of CLK, bHLH (basic helix-loop-helix), PAS (Per-Arnt-Singleminded)-A and -B, and PAC are represented as referred in Ponting and Aravind (1997), Allada et al. (1998), and Moriyama et al. (2012). **(E)** Functional domains of CYC, bHLH, PAS-A and -B, and BCTR (BMAL1 C-terminal region) are represented according to Markova et al. (2003), Kamae et al., 2010, and Uryu et al. (2013). **(G,I)** Functional domains of CRY1 and CRY2, DNA-photolyase, and FAD (flavin adenine dinucleotide) binding are represented by searching the InterPro (http://www.ebi.ac.uk/interpro/) database and as referred in Tokuoka et al. (2017). The sizes of cDNA are indicated by scales in each map **(A,C,E,G,I)**. Hyphens indicate the deleted bases in spacer and TAL RDV regions of each mutant, and identical bases are indicated by asterisks **(B,D,F,H,J)**.

To determine the role of clock genes in temperature-induced diapause, TALEN-mediated genome editing technology was used for KO silkworm *tim*, *Clk*, *cyc*, *cry1*, and *cry2* genes. Briefly, we determined the cDNA sequences containing the TALEN-target sequence of each gene in the Kosetsu strain and then designed a TALEN target for each gene (Figure 1). Thereafter, we isolated hemizygous or homozygous mutants in each gene. In *tim* mutants, a 7-base sequence was deleted, a 15-base sequence was inserted into the spacer region, and a 13-base sequence was deleted between two TALEN binding sites, designated as *∆tim2532* and *∆tim9536* (Figures 1A, B). *∆tim2532* and *∆tim9536* were considered null mutants, which could not translate full-length and lacked functional domains of TIM due to a frameshift of *tim* cDNA (Supplementary Figure S1). KO mutants of *Clk* (*∆Clk 1922* and *∆Clk7851*; Figures 1C,D), *cyc* (*∆cyc3717* and*∆cyc3718*; Figures 1E,F), *cry1* (*∆cry1_5,317*; Figures 1G,H), and *cry2* (*∆cry2_5,731*; Figures 1I,J) were generated using the same procedure. These clock gene mutants and the previously constructed *per* ^*+13*^ were used in subsequent experiments.

### Disruption of Diapause Induction in Clock Gene KO Mutants *via* Suppression of GABAergic and DH Signaling Pathways

**FIGURE 2:**
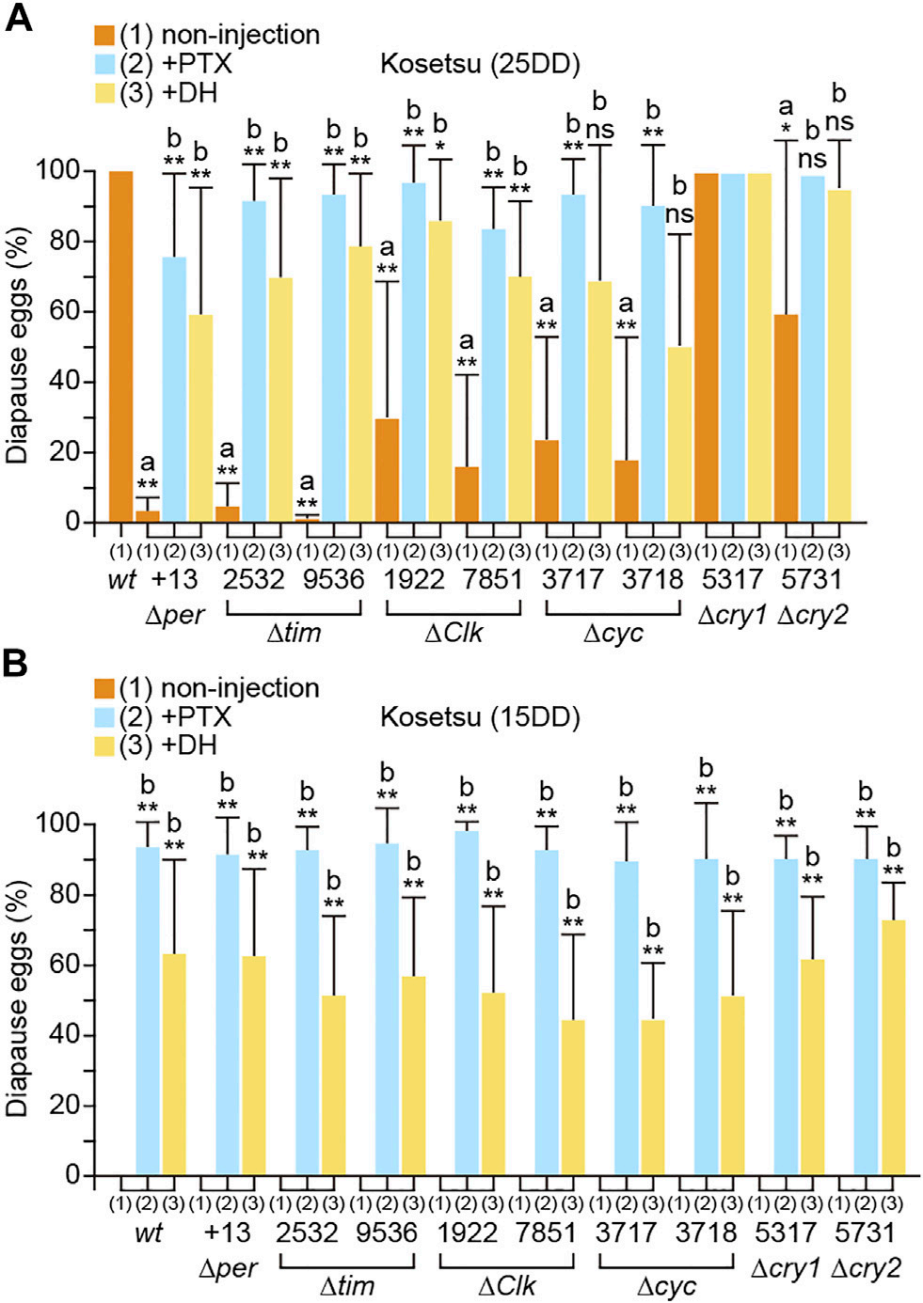
Disruption of temperature-dependent diapause induction in clock gene knockout mutants. *wt* eggs were incubated under constant darkness (DD) at 25°C [25DD] **(A)** or DD at 15°C [15DD] **(B)**. Diapause egg-inducing activities were measured in progeny eggs (non-injection). For rescue experiments, both *wt* and knockout mutants were injected with picrotoxin (PTX; 50 μg; +PTX) 1 day after pupation or with diapause hormone (DH; 100 pmol; +DH) 3.5 days after pupation, and the percentages of diapause and non-diapause eggs were determined. Each bar represents mean ± standard deviation (SD) of 20–30 animals. Statistical differences were examined by the Steel–Dwass test between each non-injected knockout mutant and *wt* **(A)** and between injected and non-injected treatments in each knockout mutant **(B)**. ns, non-significant; *, *p* < 0.05; **, *p* < 0.01.

We investigated the phenotypes of the mutants with respect to diapause occurrence (Figure 2A). The results showed that *wt* moths laid 100% diapause eggs from 25DD silkworms (silkworm resulting from eggs incubated under 25DD condition), whereas *per*, *tim*, *Clk*, *cyc*, and *cry2* KO silkworms laid varying percentages of non-diapause eggs. Particularly, knocking out *per* and *tim* remarkably reduced diapause egg-inducing activity. However, *cry1* KO did not significantly affect embryonic diapause occurrence (Figure 2A). Additionally, we examined whether each clock gene acts hierarchically upstream of the GABAergic and DH signaling pathways (Figure 2A). The pupae of the *per*, *tim*, *Clk*, *cyc*, *cry1*, and *cry2* KO mutants were injected with ionotropic GABA receptor antagonists PTX and DH since they are chemically potent diapause egg inducers (Shiomi et al., 2015; Tsuchiya et al., 2021). Both PTX and DH injection restored embryonic diapause in the pupae of *per*, *tim*, *Clk*, *cyc,* and *cry2* mutants, which was comparable to those of 25DD *wt* silkworms (Figure 2A). However, compared with 15DD *wt* silkworms (silkworms resulting from eggs incubated under 15DD condition), there was no significant difference in the percentage of diapause eggs laid by the KO mutants (Figure 2B). Furthermore, PTX or DH injection into the pupae of KO mutants promoted diapause egg-inducing activity, suggesting that the GABAergic and DH signaling pathways were retained in the KO mutants as observed in 15DD silkworms (Figure 2B).

**FIGURE 3:**
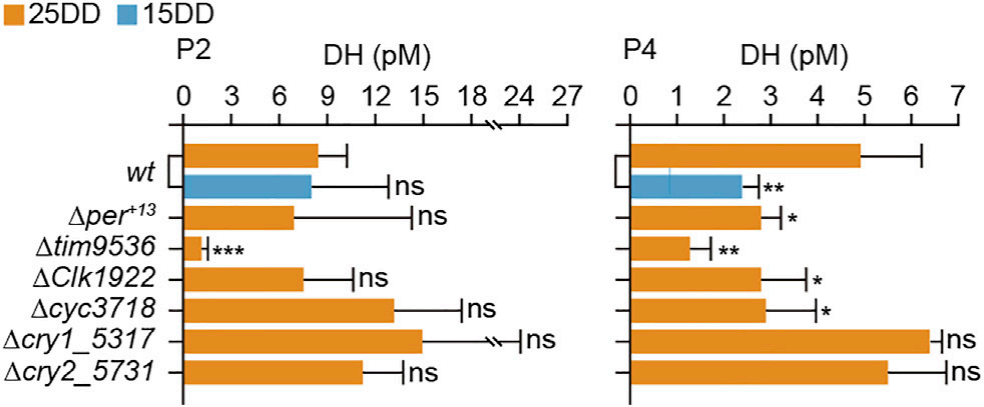
Diapause hormone (DH) concentration in hemolymph during pupal-adult development in clock gene knockout mutants. Both *wt* and knockout mutants’ eggs were incubated under constant darkness (DD) at 25°C (25DD), and only *wt* eggs were incubated under DD at 15°C (15DD). Hemolymph was collected at 2 (P2) and 4 (P4) days after pupation. Each bar represents mean ± standard deviation (SD) of five samples. Statistical differences were examined by Student’s *t*-test between *wt* at 15DD or each knockout mutant and *wt* at 25DD. ns, non-significant; *, *p* < 0.05; **, *p* < 0.01; ***, *p* < 0.001.

Moreover, the hemolymph DH levels of KO mutants were measured at 2 and 4 days after pupation (Figure 3). At 2 days after pupation (P2), the DH levels of the *per*, *Clk*, *cyc*, and *cry2* KO mutants were similar to those of 25DD and 15DD *wt* silkworms. However, there was a significant decrease in the DH level of *tim* KO silkworm. At 4 days after pupation (P4), there was a decrease in the DH levels of *per*, *tim*, *Clk*, and *cyc* KO silkworms compared with 25DD *wt* silkworms. However, the DH contents of *cry1* and *cry2* silkworms were not significantly affected (Figure 3).

### Expression of Clock Genes in Wild-Type and TRPA1 KO Mutants

**FIGURE 4:**
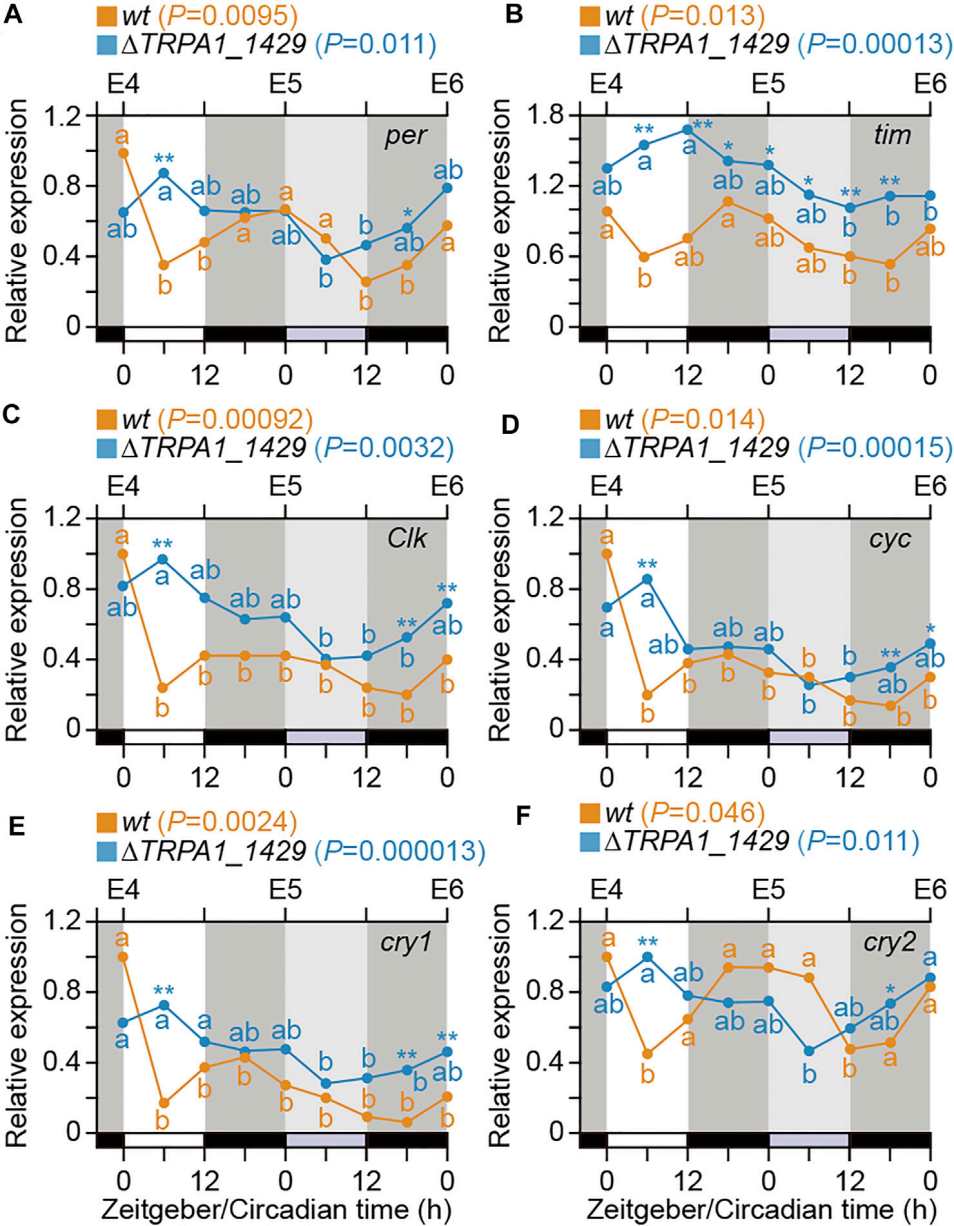
Temporal expression patterns of clock genes in eggs. Temporal profiles of clock gene expression in eggs of *wt* (orange line) and *BmoTRPA1* (*∆TRPA1_1,429*) knockout mutants (blue line) during 4–6 days (E4∼E6) after oviposition. Eggs were incubated under 12-h light/12-h dark (12L12D) conditions at 25°C and collected at 6-h intervals for a 1-day cycle (ZT0–24) and the following day in constant darkness (DD; circadian time; CT0∼24). mRNA levels of *period* [*per*] **(A)**, *timeless* [*tim*] **(B)**, *Clock* [*Clk*] **(C)**, *cycle* [*cyc*] **(D)**, *cryptochrome1* [*cry1*] **(E)**, and *crtyptochrome2* [*cry2*] **(F)** were determined by qPCR. Each value is the mean from four sets of eggs. Open bars, light; black bars, dark; gray bars, subjective day. Using one-way analysis of variance (ANOVA), *p* values are represented in each usage guide of *wt* and *∆TRPA1_1,429* in each panel. Different lower-case letters indicate values that are significantly different from each other in each *wt* (Orange) and *∆TRPA1_1,429* (Blue) [Tukey–Kramer test]. Significant differences (*wt* vs. *∆TRPA1_1,429* in each stage) are represented by an asterisk (Student’s *t*-test). *p < 0.05; **p < 0.01.

To investigate whether only *Bombyx* clock genes are the core components of the circadian clock in *B. mori* or temperature signals *via BmoTRPA1* expression also affect the expression of clock genes, we examined the expression profiles of the clock genes in *wt* and *BmoTRPA1* KO mutants during 4–6 days after oviposition (E4–E6) at 6-h intervals under the 12L12D photoperiod using qPCR (Figure 4). Generally, there were significant changes in the expression of *per* in *wt* silkworms (*p* = 0.0095, one-way ANOVA), and they exhibited a weak daily rhythm with LD and zeitgeber time (ZT). Peak expressions were observed at ZT0 and ZT24, and the lowest expression was observed at ZT6, with the rhythm persisting in DD (Figure 4A; orange line). Similarly, there were significant changes in the mRNA levels of *tim* and *cry2* (*p* = 0.013 and 0.046, respectively) in *wt* silkworms (Figures 4B, F). Although temporal changes were present in the expression profiles of *cyc* (*p* = 0.00092), *Clk* (*p* = 0.014), and *cry1* (*p* = 0.0024), the expressions were arrhythmic during the tested period (Figures 4C–E). Overall, we observed daily expressional changes of *per*, *tim*, and *cry2* in *wt* silkworms, with peak levels between ZT 16 and 24.

Moreover, although there were significant temporal changes in the expression profiles of the six genes in *BmoTRPA1* (*∆TRPA1_1,429*) KO mutants during the timeframe examined, no daily rhythm was observed (Figures 4A, B; blue lines). Additionally, a comparison of the expression levels of the genes in both *wt* and *BmoTRPA1* KO mutant silkworms showed significant differences in the expression profiles of the genes at the examined stages, especially *tim* (Figure 4; asterisk).

## Discussion

Over the years, studies have indicated the oscillating expression of core clock genes in lepidopterans, including various strains of *B. mori*, and in the monarch butterfly cell line, DpN1 (Zhu et al., 2008; Brady et al., 2021). The findings of the present study show that knocking out clock genes of *per*, *tim*, *Clk*, *cyc*, and *cry2* disrupts temperature-dependent diapause induction in silkworms. Additionally, some core clock genes, including *per*, *tim*, and *cry2*, exhibited oscillating expression during embryonic development. Together, the results suggest that the *per* and *tim* KO strains do not show circadian rhythms both in behavior and in clock gene expression (Ikeda et al., 2019; Nartey et al., 2021). We suggested that *per*, *tim*, *Clk*, *cyc,* and *cry2* are the core components of the circadian clock in *B. mori*. However, studies have revealed that the biological clocks of insects are not uniform among species. For instance, *cry1* is absent in *Tribolium castaneum* (only *cry2* exists), whereas *cry2* is present (*tim* is absent) in *Apis mellifera* (Rubin et al., 2006; Yuan et al., 2007). Therefore, further investigations are required to verify the roles of clock genes in lepidopterans.

The present study’s findings indicate that the clock genes *per*, *tim*, *Clk*, *cyc*, and *cry2* are involved in temperature-dependent diapause induction. Involvement of clock genes in photoperiodic induction of diapause has been shown in various insects, including *B. mori* (e.g., Pavelka et al., 2003; Sakamoto et al., 2009; Ikeno et al., 2010; Merlin et al., 2013; Meuti et al., 2015; Cui et al., 2021; Ikeda et al., 2021). However, the role of clock genes in temperature-dependent diapause induction has not been shown. Moreover, injection of DH or PTX induced diapause in clock gene KO mutants, and hemolymph concentrations of DH in 25DD were lower in clock gene KO mutants except for the *cry2*-KO mutant than those in *wt*. Therefore, these clock genes are involved in the upstream mechanism of DH secretion. We speculate that these genes, which probably constitute the core loop of the circadian clock, regulate temperature-dependent diapause induction as a circadian clock module (Emerson et al., 2009).

In the bivoltine strain of *B. mori*, the diapause/non-diapause phenotype is determined only during a temperature-sensitive period. This period lasts for 3 days under 25DD or 9 days under 15DD conditions, including the embryonic reversal stage, which corresponds to stages 20–23 of embryogenesis (Watanabe, 1924; Xu et al., 1995). A previous study indicated that *BmoTRPA1* is expressed in a temperature-sensitive period and acts as a thermosensitive TRP channel, which is activated at a threshold of ∼21°C, and determines progeny diapause (Sato et al., 2014). In the present study, knocking out *BmoTRPA1* disrupted the rhythmic expression of clock genes in silkworms at different stages, including the temperature-sensitive period. Moreover, studies have shown that orthologs of TRPA1 in *D. melanogaster* function in temperature control of circadian rhythm for daily behaviors (Lee and Montell, 2013; Green et al., 2015; Lamaze et al., 2017). Therefore, it is suggested that the *Bombyx* TRPA1-activated signaling pathway could be linked to circadian clock oscillation in temperature-dependent diapause induction.

Although the findings of this study show that core clock genes are involved in temperature-dependent diapause induction, the molecular mechanism of clock functions under different temperature conditions, especially 25DD and 15DD incubation conditions, is unknown. However, recent genetic and molecular studies of wild *D. melanogaster* populations revealed that polymorphisms at the *tim* locus facilitate seasonal adaptation (Tauber et al., 2007; Cai and Chiu, 2021), that is, *tim* produces four temperature-dependent splice variants in response to temperature changes. Since some splicing events generate truncated TIM proteins, this could differentially affect TIM functions, such as PER-binding and light sensitivity in the circadian clock (Cai and Chiu, 2021). Moreover, the core feedback loop of the circadian clock drives the interlocked negative feedback sub-loop. CLK/CYC activates the transcription of *vrille* (*vri*) and *Par domain protein 1ε* (*Pdp1ε*) and activates the transcription of *clockwork orange* (*cwo*), which regulates the amplitude of *per* and *tim* mRNA oscillations (Tomioka and Matsumoto, 2015). Overall, we speculated that there are differences in the expression levels, selective splicing, and functions in clock genes and/or clock-related genes under 25DD and 15DD incubation conditions, which could affect clock gene oscillations, such as stability and amplitude for diapause determination.

A previous study demonstrated that knocking out *BmoTRPA1* can disrupt the TRPA1-activated pathway, which could influence temperature-dependent diapause induction and initiate photoperiodic diapause induction (Yokoyama et al., 2021). Therefore, it is suggested that the *wt* Kosetsu strain potentially possesses a signaling pathway involved in photoperiodic diapause induction that might be canceled by the strong linkage of *BmoTRPA1*-activated and DH signaling pathways. Consequently, the progeny diapause may be dependent on the photo- or scotophase length in *BmoTRPA1* KO mutants. Therefore, we speculate that clock genes are engaged in both photoperiodic and temperature-dependent diapause induction and that the circadian clock is involved in the selection and integration of several environmental signals.

A previous study found that the expression of the plasma membrane *GAT* gene was 10–100-fold higher in the pupal brain–subesophageal ganglion complex under 25DD incubation condition than that under 15DD incubation condition (Tsuchiya et al., 2021). Under 25DD conditions, the female adults of the *GAT* KO mutant laid mostly non-diapause eggs. Therefore, we conclude that the GABAergic signaling system may be influenced by the temperature-dependent expression of the plasma membrane *GAT* since the plasma membrane *GAT* actively transports GABA from the synaptic cleft back into presynaptic neurons and glial cells to terminate GABA-stimulated responses (Tsuchiya et al., 2021). We demonstrated that clock genes are involved in temperature-dependent diapause induction upstream of GABAergic and DH signaling pathways. Therefore, clock genes may influence GABAergic signals directly and indirectly by regulating *GAT* expression, thereby inducing embryonic diapause in progenies. Overall, the findings of this study provide an excellent model for investigating relationships among the circadian clock, temperature, and photoperiodic response.

## Data Availability

The original contributions presented in the study are included in the article/Supplementary Material, further inquiries can be directed to the corresponding author.
