# Supplementary material for: Circadian Clock Genes Regulate Temperature-Dependent Diapause Induction in Silkworm Bombyx mori
Source: Front Physiol. 2022 Apr 27;13:863380. doi: 10.3389/fphys.2022.863380 (PMC9091332; doi:10.3389/fphys.2022.863380)
Supplement: Supplementary file 1 [file Table1.pdf]

Supplementary Table S1

List of primers*	
a. For KO screening	
1. *timeless* (*tim*)	F: 5′-GAGCAATACCTCTCCGCCGGATCG-3′
	R: 5′-TGAAAACGTTACCGATTTCGCTTC-3′
2. *Clock* (*Clk*)	F: 5′-CAATGTTCTCTAAAGGTTTGGGAATGG3′
	R: 5′-TCTGAAGTCTAGTCTTCCAGTAACAAC-3′
3. *cycle* (*cyc*)	F: 5′-GGAACTGGAAGCGCTTATGATGACGAC-3′
	R: 5′-ATTTTTGACCGAAGCCGAGACGTAAAG-3′
4. *cryptochrome1* (*cry1*)	F: 5′-AAGCACGCAATCACACATTGATAAGTGCTC-3′
	R: 5′-AGGTGTCATTAGGACTATCGTCTATCTTCG-3′
5. *cryptochrome2* (*cry2*)	F: 5′-GAGCCAAACGGGACTATCACCATACTTAAG-3′
	R: 5′-GGCATCAATCCAAGGGTATCCGGTTTGCCC-3′
b. For qPCR	
1. *period* (*per*)	F: 5′-TTGAGCAGCCGTCACTAGTG-3′
	R: 5′-TGTCTCTTCACCGGAACTGC-3′
2. timeless (*tim*)	F: 5′-CGGTACGCAAAGAGATGACG-3′
	R: 5′-GGCTGTATGTCGCCTTCCTC-3′
3. *Clock* (*Clk*)	F: 5′-TTACCTTTGCCGCCTCTACC-3′
	R: 5′-TTCACCTGTCGCAACTCCTC-3′
4. *cycle* (*cyc*)	F: 5′-CAACAACTACAACCACGGCG-3′
	R: 5′-ATCGGTATCATGGCGCTCAG-3′
5. *cryptochrome1* (*cry1*)	F: 5′-AGGGACGAGAGCGTGAAGAC-3′
	R: 5′-TTCGCTTTGATGACGGTGTC-3′
6. *cryptochrome2* (*cry2*)	F: 5′-CTGTGCTGCGACAAGAAACC-3′
	R: 5′-TTGCCCATTTGCCCATTTCG-3′
7. *rp49*	F: 5′-GCATCAATCGGATCGCTATG-3′
	R: 5′-CGTAACCAATGTTGGGCATC-3′

*F; Forward primer, R; Reverse primer.
