# Supplementary material for: Circadian Clock Genes Regulate Temperature-Dependent Diapause Induction in Silkworm Bombyx mori
Source: Front Physiol. 2022 Apr 27;13:863380. doi: 10.3389/fphys.2022.863380 (PMC9091332; doi:10.3389/fphys.2022.863380)
Supplement: Supplementary file 2 [file DataSheet1.pdf]

A *timeless (tim)*

Δ*tim2532* (121 a.a)

```
MIQFIALLYKNQHAVTLHKLLNLWLESSLSESSEDNESNTSPPDRGSDDSSPILTSDPTS
DSSDTGGSGKSNDEPNSVNNGWDASSVNNTSENGSQTSQTHNDSEIIFLCGKMRKILQRAP*
```

Δ*tim9536* (114 a.a)

```
MIQFIALLYKNQHAVTLHKLLNLWLESSLSESSEDNESNTSPPDRGSDDSSPILTSDPTS
DSSDTGGSGKSNDEPNSVNNGWDASSVNNTSENGSQTSQTHNEGKMRKILQRAP*
```

B *Clock (Clk)*

Δ*Clk1922* (38 a.a)

```
MEDDGDDKDDTKRRTRNLSEKKRRDQFNMLVNELSSMV*
```

Δ*Clk7851* (40 a.a)

```
MEDDGDDKDDTKRRTRNLSEKKRRDQFNMLVNELSSMEDG*
```

C *cycle (cyc)*

Δ*cyc3717* (180 a.a)

```
MADFIDEASTSQRGHANPSAIQAYEMTPEGGVGLGGACADSAGALITPHPPLHHPVPQTSQQ
LHHDPRKTKPNHYVPENYEISACDSQRQSPHGHTPRTRTNSTRKRKPSSYGTGSAYDDDEED
SRSTTTTTTATTRGTPDKKQNHSEIEKRRRDKMNTFISELSAMIPMCGAWLGSWTS*
```

Δ*cyc3718* (212 a.a)

```
MADFIDEASTSQRGHANPSAIQAYEMTPEGGVGLGGACADSAGALITPHPPLHHPVPQTSQQ
LHHDPRKTKPNHYVPENYEISACDSQRQSPHGHTPRTRTNSTRKRKPSSYGTGSAYDDDEED
SRSTTTTTTATTRGTPDKKQNHSEIEKRRRDKMNTFISELSAMIPMCGDGSEAGQADSPPHG
RSTLEDGARRALGLPLDGPALSHLPH*
```

D *cryptochrome1 (cry1)*

Δ*cry1_5317* (56 a.a)

```
MLGGSVLWFRHGLRLHDNPSLHSALEETSALSFPFSFLTGKLLERKWSGTTECGTC*
```

E *cryptochrome2 (cry2)*

Δ*cry2_5731* (301 a.a)

```
MSAAPETLPPPSAQAHTPARPTHMSAPRRTPGKHTVHWFRKGLRIHDNPALREGIIDAVTFR
CVFIIDPWFASSSNVGINKWRFLLQCLEDLDKSLKKLNSRLFVVRGQPADALPKLFREWGTT
ALTFEEDPEPYGRVRDHNIISKCREVGITVTSRVSHTLYKLDKIIERNGGKAPLTYHQFQAL
IASMPPPPPAEVTITPQMLNGATTPITDNHDDRFGVPTLEELGFETEGLKPPIWIGGESEAL
ARLERHLERKAWVASFGRPKMTPQSLLASQTGLSPYLRFGCLSIVLLSIDGIV*
```

Supplementary Figure S1. Putative amino acid sequences of clock gene knockout mutants. Δ*tim2532* and Δ*tim9536* (A), Δ*Clk1922* and Δ*Clk7851* (B), Δ*cyc3717*, Δ*cyc3718* (C), Δ *cry1_5317* (D), and Δ*cry2_5731* (E) produce truncated proteins, which consist of 121, 114, 38, 40, 180, 212, 56, and 301 amino acids, respectively.
